# The Third South Midland Field Ambulance

**Published:** 1913-06

**Authors:** A. W. Prichard

**Affiliations:** Late Officer Commanding.


### THE THIRD SOUTH MIDLAND FIELD AMBULANCE.

BY

Lieut.-Colonel A. W. PRICHARD, V.D.,

*Late Officer Commanding.*

---

In the autumn of 1907, while the Volunteers were still in existence and not in a very satisfactory condition as regards numbers and efficiency, the formation of the Territorial Force in their stead was one of the topics of the day. I was then Brigade-Surgeon of the Portland Volunteer Infantry Brigade, and hearing that the Director-General of Medical Services was about to address the medical men of Birmingham with reference to the raising of R.A.M.C. units in the grouped regimental district in which both Birmingham and Bristol are situated, I urged the claim of Bristol to take its share, and argued that with its excellent hospitals, medical societies, University College and Medical School, to say nothing of the keenness with which men had joined the Artillery, Engineer and Infantry Volunteers, it could not well be left out. It was therefore decided by the War Office that of the three field ambulances and two mounted brigade field ambulances that serve each division, one field ambulance should be allocated to Bristol, and I was asked to raise it and take command.

On January 11th, 1908, a public meeting, under Colonel Savile, C.B., and a meeting of medical men of Bristol and the neighbourhood, under Dr. Michell Clarke, were called, and at both of them Colonel Russell of the War Office was present, and explained the organisation of the medical services. A resolution was passed approving of the steps that had already been taken, and I had the satisfaction of receiving the names of several officers who were willing to serve under me.

As at that meeting of the profession the real start was made, it may be not unseemly if I give details of the history of the Field Ambulance for the five years that I have had the pleasure and honour of being in command.

No real recruiting was allowed before April 1st, 1908, when the transfer of the Volunteers to the Territorial Force was to take place. I could only get promises of names. King Edward was to come on the 9th of July to open the docks at Avonmouth, and until I had eighty men—a third of my establishment—the unit could not be recognised, and could not be clothed by the County Association. With the assistance of Major Young, many meetings were held, and the work of organisation made good progress. Our efforts were ably supported by Dr. Rogers, Dr. Green and Mr. Lambert (quartermaster), who had expressed their desire to take commissions in the new Field Ambulance. Colonel Woodward, of the Bristol Rifles, kindly allowed us part of his premises as head-quarters, and drills, lectures and officers' rides were begun. The head master of the Grammar School let us drill on the Grammar School ground, and we got our eighty men just in time to clothe them and drill them enough to fall in and help line the streets on the occasion of the King's visit. That was the first public appearance of the Bristol Territorial R.A.M.C.

Recruits came in rapidly, and in August we went to Swanage to camp for a week as a medical corps attached to the Gloucester and Worcester Brigade. We were very new to the work, and there were difficulties to be overcome, the chief of these being with the heavy transport and the railways. The second week of that fortnight was at Perham Down, Salisbury Plain, where we were separated from the Gloucesters and brigaded with the 1st and 2nd Field Ambulances from Birmingham, whose acquaintance we then made for the first time. During this week we left camp early one day and did not return until six o'clock next evening, bivouacking at night in a field with many other troops on the side of the Avon above crossing "A." After a long field-day and a march past on Knighton Down, we had a ten-mile march back to camp.

Our first camp over, after a rest for a month or so, we began lectures and drills. We started a band, and had a first church parade of our own, which General Raitt attended, with the result that recruits came in willingly, and by the end of the winter we had recruited up to full strength, both in officers and men, a result that I believe no other Field Ambulance in England that had not had a bearer company of the old Volunteers to start it had achieved. We have been above strength ever since.

On April 1st, 1909—our first birthday—my officers were Major Young and Lieutenants Rogers, Mather, Green, Lavington, Moxey, Coleridge, Corfield and Lambert. We were busy with lectures, company and stretcher drills in the Grammar School ground, bugle practice, and signalling, and in July had the satisfaction of receiving from the medical profession of Bristol, in recognition of our efforts, a drum-major's staff, the presentation being made at the Drill Hall by General Raitt. That summer the fortnight's camp was again at Swanage, and a very enjoyable camp it was. We went down nearly full strength with all transport complete. We had the best position in the Brigade Camp, on top of a hill where there was welcome shade of trees. At the bottom of the hill was a large, flat meadow, which served the purpose of parade ground, cricket ground, and riding school. Looked at from the point of view of a pleasurable outing, at a pleasant seaside place, with bathing parades, cricket and country route marches, the camp of 1909 was a great success.

After this camp the officers decided to run their own mess. A business arrangement was entered into among the officers, so that all tent equipment and table necessaries should belong to themselves. All this has to be taken to camp every year, and a mess president is appointed to engage a cook and waiters and be responsible for ordering the necessary provisions. We have found this plan to work very well.

Nothing of great importance occurred during the next winter. All the officers worked well; they had to pass their examination for certificate B, and things went very smoothly, except as regards the difficulty of getting our own head-quarters. Next summer, 1910, on Salisbury Plain a very pleasing honour was conferred upon the Bristol unit during this camp. A complete Field Ambulance of regulars was in training at Tidworth, and were to be inspected by the Duke of Connaught. It so happened that upon the day of the royal inspection I had been invited by the commanding officer of the regulars to bring my territorial corps from Perham Down to watch the work of the regulars. The Bristol unit was halted some little distance away from the scene of the operations, and saw the Duke arrive with his staff. His Royal Highness had been upon the ground but a few minutes when he observed the Bristol men in the distance, and he at once sent an officer with a command that I should bring my men to the ground where he was holding his inspection. We were allowed to watch the full routine of the work properly done, and afterwards greatly honoured at being ordered to march past, our band playing the R.A.M.C. march.

In 1911 the officer commanding the Bristol Rifles found that it was not convenient to harbour us any longer, and we had to seek shelter elsewhere. We therefore engaged the Hannah More Rooms in Park Street, where there were rooms for lectures, quartermaster's stores, sergeants'. mess, and a small hall for drill. Our head-quarters at Kingsdown were being built. We tried one evening parade on Brandon Hill, but it was impossible to carry it through with decorum, owing to the huge number of children that were attracted by the bugles. This year we went full strength to Towyn with the Warwick Brigade and the other Field Ambulances. It was lucky it was a dry fortnight, as our camp was on ground that wet weather would have turned into a bog. We had no rain until the day of our departure, and that was a memorable occasion, as owing to the coal strike no trains were running, and we thought we should have to stay longer than we liked. However, the men were allowed by the railway companies to come away on the afternoon of the day fixed, and it took twelve hours, nearly half of which was in a train with no lights, to get home. The horses did not reach home until some days later. We came back to our new head-quarters, at the back of the "Montague," Kingsdown, which were nearly ready.

The chief event of the winter was the opening of the new head-quarters at Kingsdown, on November 25th, by Lord Beauchamp. The President of the County Association attended, accompanied by Sir Arthur Anstice, Colonel Savile, Captain Colchester Wemyss, and many of the officers of the territorial units. All who had been invited to attend made a tour of inspection, and expressed considerable satisfaction at the arrangements that had been carried out. Some old coach-houses had been re-roofed and turned into commodious wagon sheds for the ambulances, with a covered way for them down into the drill yard; another shed had been converted into a heavy storeroom, and a three-stalled stable had been fitted up as an excellent harness-room. A new lecture-room, sergeants' mess and large drill hall had been built, and the interior of Colston Fort House itself had been re-arranged for officers' room, orderly room, quartermaster's stores, and various offices. Lord Beauchamp performed the opening ceremony, and the function passed off very pleasantly. A fund was started to supply proper gymnastic appliances, and within two months we had a good gymnastic class at work.

The year 1912 will be remembered for two reasons—the week-end Whitsuntide camp and the summer drenching on Salisbury Plain. At Whitsuntide a week-end camp was held at Tockington, and four fine days were much enjoyed. The August camp was very different. It was situated at West Down South, a most exposed place and seven miles from anywhere. We marched in dry, and then had no day without rain. However, a great deal of real training was done. We were with the other two Ambulances, two infantry brigades, and many other troops. Much interest was taken in the flying from Larkhill, which took place whenever the weather made it possible; and the day which the men enjoyed most was a route march to Stonehenge and Amesbury. We took meat and vegetables and stopped for two hours in the middle of the day, made fires and cooked it, and on the way back halted at Stonehenge and saw many aeroplanes flying close over us.

During this camp all field drill was by whistle and signal, no general word of command ; and a complete system of competitions for prizes in the different classes, such as company drill, stretcher and wagon drill, signalling, bugling, and the grooming and keep of harness and horses, was carried out.

I was greatly pleased at a sentence in the Special Sanitary Officer's (Southern command) report : "This is the unit with the best arrangements for sanitation in camp. A great deal of time and trouble is expended on it, and but for the deficiencies of the contractor it may be considered a model to others." The "deficiencies of the contractor" refers to the slowness of carting the refuse away.

At home during the last winter special attention has been paid to the social side of Territorial life. A great many 1908 and 1909 men signed on for four years' service, and to encourage some of them to re-engage as their time expired and to bring other friends in, a series of smoking concerts and a Christmas entertainment were held, with, I hope, a good result. My time for retirement came, and my last parade was a church parade at the Cathedral on February 23rd, and I took leave of the corps with many feelings of regret at a well-attended R.A.M.C. dinner on March 5th.

In conclusion, I wish to say that I do not take the credit of the success of the undertaking to myself. I have omitted in my remarks any reference to the N.C.O.'s, who have, many of them, given up a great deal of time after working at their various occupations all day to, first of all, getting their promotion by examination—and that examination no light one—and afterwards carrying out their duties as soldiers should. To them and to the whole of the officers the credit is due. Mess dinners (three or four in the year), officers' rides, officers' meetings, and camp have united us together like a happy family, and made the time to me very pleasant ; and I am sure the present commanding officer, Lieut.-Colonel James Young, will keep up and enhance the prestige which I believe the 3rd South Midland Field Ambulance at present holds.

The following is a list of the officers up to April, 1913 :—Lieut.-Colonel A. W. Prichard, 1.4.08 ; Lieut.-Colonel J. Young, 1.4.08 ; Captain B. M. H. Rogers, 1.4.08 ; Captain J. S. Mather, 1.4.08 ; Captain T. Green, 1.4.08 ; Captain P. Moxey, 1.4.08 (transferred to the 6th Batt. Glo'ster Regiment for one year and then rejoined the Field Ambulance) ; Captain A. Coleridge, 23.3.09 (transferred to 4th Wessex R.A.) ; Captain C. C. Lavington, 15.1.09 ; Lieut. C. Corfield, 18.3.09 (attached to S.M.F.A.) ; Lieut. C. E. K. Herapath, 1.4.11 ; Lieut. G. Scott Williamson, 1.11.09 (transferred from York Mounted Brigade F.A.) ; Lieut. C. F. Walters, 18.1.11 (transferred from 2nd Southern General Hospital, retired) ; Lieut. and Quartermaster H. Lambert, 1.4.08 ; Chaplain Rev. Canon J. G. Alford, V.D., 1.1.10.

